# Food Safety: Pathological and Biochemical Responses of Nile Tilapia (*Oreochromis niloticus)* to Parasitological Infestation and Heavy Metals Pollution in Aquaculture System, Jeddah, Saudi Arabia

**DOI:** 10.3390/ani15010039

**Published:** 2024-12-27

**Authors:** Muslimah N. Alsulami, Sarah Khaled Baowidan, Rabab M. Aljarari, Haleema H. Albohiri, Samar A. Khan, Elham Ali Elkhawass

**Affiliations:** 1 Department of Biology, College of Science, University of Jeddah, Jeddah 21589, Saudi Arabia; mnal-sulami@uj.edu.sa (M.N.A.); sbaowidan0001.stu@uj.edu.sa (S.K.B.); rmaljerary@uj.edu.sa (R.M.A.); hhalbahiri@uj.edu.sa (H.H.A.); sakhan@uj.edu.sa (S.A.K.); 2 Department of Zoology, Faculty of Science, Suez Canal University, Ismailia 41522, Egypt

**Keywords:** parasites, tilapia, *Oreochromis niloticus*, metals pollution, antioxidant responses, histological alterations

## Abstract

Objective: The study aims to assess the overall safety of cultured tilapias in Jeddah City, Saudi Arabia by assessing the impact of infection and anthropogenic pollution on farmed tilapias based on fish sex, body weight, length, and heavy metals contamination. Materials and methods: A total of 111 fish were collected from an aquaculture farm in Hada Al-Sham, Jeddah, Saudi Arabia. Physicochemical parameters of water from the culture system were evaluated. Both ecto- and endoparasites were checked. Haematological, biochemical and histopathological investigations were evaluated. In addition, heavy metals, namely, cadmium (Cd), chromium (Cr), copper (Cu), nickel (Ni), lead (Pb), and zinc (Zn) were evaluated in different fish tissues and water samples from the aquaculture system. Results: The study revealed stressed aquaculture system. Tilapias were infested by both ectoparasites including *Trichodina*, *Icthyophthirius multifiliis*, *Dactylogrus*, and *Cichlidogyrus*, and endoparasites as *Icthyophonus hoferi*, the nematode *Capillaria* and coccidian protozoa. The study showed that male tilapias had greater infestation rates than females and longer and heavier male fish tended to be more susceptible to *Dactylogyrus* infection. Infected fish showed altered biochemical markers with subsequent increases in inflammatory and oxidative stress markers. The post-mortem lesion in the skin, gill lamellae, intestine, spleen, and liver showed significant pathological remarks. All investigated fish tissues revealed higher rates of heavy metals bioaccumulation compared to the surrounding waters. On the other hand, infected Nile tilapia tissues showed higher rate of metals accumulation compared to non-infected ones. Metals accumulated at a higher rate in the liver followed by kidney, intestine, gills, and muscles, respectively. Conclusions: This study is recognized as the first to address the food safety of farmed tilapias in Jeddah, Saudi Arabia. The results emphasized a significant relation between parasites and heavy metal in disrupting fish defense systems and harming fish’s physiological homeostasis and the histological state of tissues. The parasitized and polluted farmed fish pose health risk to humans due to possible zoonosis from parasitic infections and its subsequent bacterial infections with long-term exposure to toxic chemicals. Addressing the need for a combination of improved aquaculture practices, and stringent regulatory oversight.

## Simple Summary

Fish culture is an important sector in securing food supply for human, however, potential hazards as biological and chemical contamination is a challenge. This study is recognized as the first to address the food safety of farmed tilapias in Jeddah, Saudi Arabia. The study aims to assess the overall safety of cultured tilapias in Jeddah City by assessing the impact of infection and anthropogenic pollution on tilapias based on sex, body weight, length, and heavy metals contamination. The study revealed stressed aquaculture system as tilapias were infested by both ectoparasites and endoparasites. Infestation was found to be sex, length and weight dependent. Male tilapias had greater infestation rates than females and longer and heavier male fish tended to be more susceptible to infection. All investigated fish tissues revealed higher rates of heavy metals bioaccumulation compared to the surrounding waters. Infected fish showed increases in inflammatory and oxidative stress markers. The results emphasized a significant relation between parasites and heavy metal in disrupting fish defense systems and physiological homeostasis. The study emphasizes that parasitized and polluted farmed fish pose health risk to humans. Addressing the need for a combination of improved aquaculture practices, and stringent regulatory oversight.

## 1. Introduction

One of the primary sources of animal protein, omega-3 and omega-6 fatty acids, and lipid-soluble vitamins in the diets of humans is fish [1,2]. Aquaculture is a quickly rising sector of the global livestock industry and it is presently meeting the increasing demand for fishery products and greatly increasing fish output globally [3,4,5].

Nile tilapia (*O. niloticus*) is a freshwater cichlid that is native to the Nile basin. It is considered to be one of the most common freshwater fish [6]. Worldwide, tilapia is farmed in more than 135 nations. Due to its inexpensive price, rich flavor, and high nutritious content, it is enjoyed in many different countries. In the upcoming years, there will likely be a further increase in the demand for tilapia due to factors like population growth, rising earnings, and growing knowledge of the health benefits of eating fish [7].

Heavy metals are non-biodegradable that may cause toxic pollution in the aquatic environment worldwide if they surpass certain thresholds [8,9]. Fish have a potential for metal poisoning as they accumulate heavy metals from the surrounding water and store them in varying levels in different tissues [10]. Metals in the aquatic systems can enter the bloodstream of fish and progressively build up in their tissues where they can be eliminated by liver or sustained in fish tissue and reach consumers through the food chain [11,12,13]. Various metals (As, Cd, Fe, Cu, Cr, Hg and Pb) are major factors of causing oxidative stress in fish. The solubility, and complexation of metals play a key role in determining the toxicity of metals in aquatic environments [14,15]. Heavy metal accumulation in fish is more likely to produce reactive oxygen species (ROS), which can alter the morphology and biochemistry of their tissues [14,16].

In both aquaculture and wild fisheries, fish are highly susceptible to a variety of diseases. Moreover, a variety of parasites have been linked to zoonotic illnesses worldwide even in seemingly healthy fish [17,18]. Pollution and parasitism are related, and parasites can serve as bioindicators for heavy metal contamination [18,19]. Previous reports have addressed the ability of some parasites to accumulate heavy metal from their hosts, such as Acanthocephalans, cestodes, and parasitic nematodes [20,21]. Parasites are thought to be particularly vulnerable to heavy metal pollution. Fish parasites have high potential to accumulate metals in higher concentration than their hosts and even when metals are in low concentrations [22,23]. Moreover, parasites are found to be sentinel species that tolerate high concentrations of heavy metals in their habitat [24].

Asia demonstrated a low clustered prevalence rate of parasitic infection in fish (0.10; 95% CI: 0.04–0.18), indicating insufficient attention to public health issues in certain countries within the region [25]. According to the ministry of Environment, Water, and Agriculture, the fish farm production in Saudi Arabia surged by 56.4%, reaching a record 140,000 tons in 2023 aiming to reach annual fish consumption of 13 kg per capita. The favored cultured species in Saudi Arabia include Nile tilapia, sea bass, dentex, and shrimp [26].

Food security, public health, and sustainable aquaculture is crucial for developing effective fisheries management, and public health policies. Toxicological, and parasitological studies deliver comprehensive insights into the health of aquatic organisms and their ecosystems. The present study aims to assess the food safety and quality of the Nile tilapia (*O. niloticus*) supply in Jeddah City, Saudi Arabia based on the water quality of the aquaculture system and fish wellbeing. The study investigates different physicochemical parameters of the culture water, parasitic infections and their prevalence in farmed tilapias in relation to different factors such as fish sex, body weight, and length. The study also assesses the alteration in the haematological, biochemical and histopathological parameters in farmed tilapias due to infection and heavy metal pollution.

## 2. Materials and Methods

### 2.1. Study Area and Fish Sampling

A total of 111 Nile tilapia (*O. niloticus*) were collected from an aquaculture farm at the Hada Al-Sham (about 117 km northeast of Jeddah city in the western province of Saudi Arabia) at 21°47′48″ N and 39°41′0″ E. Collection took place from November 2022 to November 2023. The fish were transferred alive to the lab and housed in water-filled, aerated vessels till the examination.

### 2.2. Physicochemical Properties of Water

Duplicate water samples were taken at different depths and dates from the farm. Water’s physicochemical characteristics were assessed. Water temperature, electrical conductivity (EC), salinity, total dissolved solids (TDS) were measured using portable temperature conductivity meter (HI 9635, Hanna Instruments Inc., Smithfield, RI, USA), while hydrogen ion concentration (pH) was measured using portable pH meter (HI 8543, Hanna Instruments Inc., Rhode Island, USA). Also, ammonia, nitrate, nitrite, total alkalinity, total hardness, phosphate, sulfate, iron, and chlorine using a spectrum photometer according to [27]. Heavy metals (Cd, Cr, Cu, Ni, Pd, and Zn) were also detected in the water samples and expressed in mg/L. Water samples were mixed with 1% HNO_3_ at 4 °C and analysed for metals using Inductively Coupled Plasma/Mass Spectrometry (ICP/MS) according to standard procedures [27,28].The mean of water physicochemical parameters compared to the standard values provided by the World Health Organization (WHO) [29].

### 2.3. Fish Health Assessment

#### 2.3.1. Fish Physical Examination

During examination, fish sex was determined by genital papilla. Then, the fish weight was measured, and split into four groups: I (70–100 g), II (110–200 g), III (210–300 g), and IV (>310 g). The fish length (from nostril to the end of caudal fine) was divided into three groups: I (<18 cm), II (18–28 cm) and III (>28 cm) [30,31].

#### 2.3.2. Parasites Identification

Fish were examined externally for any visible parasites, and a scrap from the body mucus over was checked for ectoparasites under light microscope [32]. Giemsa stain was used for protozoan parasites, and carmine stain was used for nematodes, cestodes, and trematodes [33]. Furthermore, the fish were dissected, and the kidney, liver, spleen, intestines, muscles, and gills were examined for any parasites [34]. 

#### 2.3.3. Haematological and Biochemical Analyses

Shortly after fish sampling, blood samples were drawn from the caudal vein and two aliquots were used. One aliquot was used for the haematological parameters as total leukocyte counts (TLC), differential leukocyte counts (DLC), red blood cells (RBC), haemoglobin content (HGB), hematocrit (HCT), mean cell haemoglobin (MCH), mean corpuscle volume (MCV), mean corpuscular, mean cell haemoglobin (MCH), haemoglobin concentration (MCHC), and platelet counts (PLT) using automated hematology system XE-2100 (TOA Medical Electronics, Kobe, Japan) [35,36].

The second aliquot was collected in dry, clean tubes (without anticoagulants) for the biochemical parameters and carried out with automated serum biochemistry analyzer (DYNEX DSX best 20000 Automated ELISA System, DYNEX Technologies, Chantilly, VA, USA) using commercially available kits. Parameters included alanine aminotransferase (ALT) (Cat. No. MBS2800393), aspartate aminotransferase (AST) (Cat. No. MBS1601734), alkaline phosphatase (ALP) (Cat. No. MBS1601674), and tumour necrosis factor-alpha (TNF-α) (kit number: MBS704369); superoxide dismutase (SOD) (Cat. No. MBS705758), and catalase (CAT) (Cat. No. MBS705697). Malondialdehyde (MDA) was also detected using fish malondialdehyde ELISA kit (Cat. No. MBS1601664).

#### 2.3.4. Histopathological Studies

Fresh tissue samples from the skin, gills, liver, spleen, and intestine were preserved in 10% neutral-buffered formalin, dehydrated, cleaned with xylene, embedded in paraffin blocks, and sectioned at 4–6 μm thick and stained with haematoxylin and eosin (HE) [37].

#### 2.3.5. Heavy Metal Bioaccumulation in Fish Tissue

Heavy metals assessment was done according to [38,39]. Fish organs (liver, kidney, spleen, intestine, and gills) were weighed (one gram each) and acid digested for Cd, Cr, Cu, Ni, Pd, and Zn estimation via Flame Atomic Absorption Spectroscopy (FAAS). The final concentration was expressed in mg/g.

### 2.4. Statistical Analysis

All data was investigated using MS Excel and SPSS software (version 22). Chi-square or Fisher exact test were used to analyze the infection frequency by sex, weight, and length. Haematological and biochemical parameters in both uninfected and infected fish were compared using the independent *t*-test. Two-way ANOVA post hoc Tukey was applied to quantify the statistical difference between the heavy metals’ levels in various fish organs. Pearson’s correlation coefficient used to evaluate the associations between physio-chemical parameters and heavy metal concentrations in both water and infected tissues. Results are expressed as mean ± standard deviation (SD). Statistical significance was defined as a *p*-value of less than 0.05. All the graphs were performed using GraphPad Prism software version 10.2.3 for Mac (GraphPad Software, San Diego, CA, USA).

## 3. Results

### 3.1. Analysis of Aquaculture Water Quality

Water nitrate and salinity show normal limits. However, TDS, total hardness, total alkalinity, ammonia, nitrite, and phosphate show a substantial increase compared to the standard values as reported by WHO as shown in Table 1. The metals Cu and Zn occur at the higher boarder of the limits set by standard organizations (WHO). Moreover, the heavy metals Cr, Cd, Pb, and Ni are recorded in a higher threshold than acceptable universal thresholds by WHO.

The Pearson correlation between physio-chemical parameters and water-heavy metals (Supplementary S1) shows a significant positive correlation between salinity versus electric conductivity, TDS, Ammonia, nitrate, Nitrite, total alkalinity, Total Hardness, phosphate, Sulfate, and Iron (r = 0.88, 0.88, 0.83, 0.85, 0.83, 0.86, 0.88, 0.86, 0.87, and 0.84, respectively) and a significant positive correlation between Nitrite versus electric conductivity, TDS, Ammonia, and nitrate (r = 0.94, 0.94, 0.94, and 0.95, respectively). Moreover, there is a significant positive correlation between Nitrite versus Total Alkalinity, Total Hardness, Phosphate, Sulfate, Iron, and Chlorine (r = 0.97, 0.94, 0.95, 0.94, 0.97, respectively). In addition, there is a significant negative correlation between Zn- Pb (r = −0.86) and Zn-Cu (r = −0.97). However, there is a significant highly positive correlation between Pb-Cu (r = 0.95), and Pb-Cr (r = 0.81). While, there is no significant relation between the association of other metals in the water.

### 3.2. Fish Parasites Assemblage

**Figure 1:**
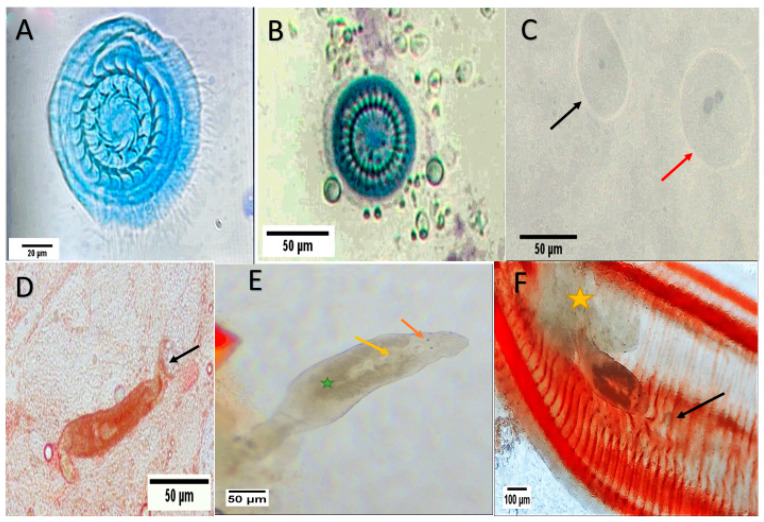
Microscopic examination of external pathogen showing: (**A**) *Trichodina heterodentata* with 24 denticles; (**B**) *Trichodina truttae* with 28 denticles; (**C**) *Ichthyophthirius multifiliis*, red arrow: trophont and black arrow: theront; (**D**) *Dactylogyrus* spp. (arrow: posterior haptor); (**E**) *Cichlidogyrus* attached to gills (orange arrow: eyes; yellow arrow: male copulatory organ; green star: vitellaria); (**F**) *Cichlidogyrus* attached to gill filaments (yellow star: thick slime; black arrow: protozoan *Trichodina*).

The parasitological examination of the external of infected tilapias shows two *Trichodina* species; *T. heterodentata* and *T. truttae* (Figure 1A,B). The trophont and theront of *I. multifiliis* were detected as tiny dots (up to 1 mm) on the body surface and gills (Figure 1C). Two genus of monogeneans worms are collected; *Dactylogyrus* spp. with two pairs of eye spots on the anterior, and a haptor at the posterior end, and one transverse bar (Figure 1D,E) and *Cichlidogyrus* spp. with two pairs of eye spots and two pairs of anchors (Figure 1F,F). 

**Figure 2:**
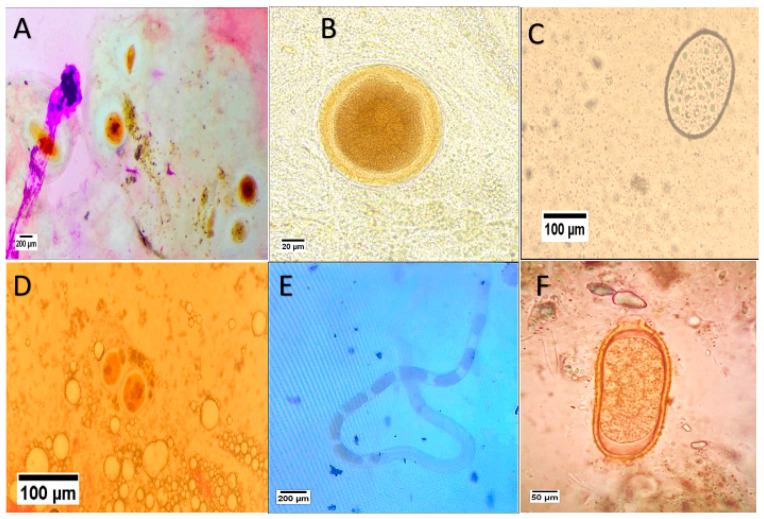
Microscopic examination of the internal pathogen showing: (**A**) *Ichthyophonus hoferi* in squash of kidney showing resting spore/schizont ×40; (**B**) *I. hoferi* showing resting spore/schizont with fibrotic capsule in kidney ×400; (**C**) *I. hoferi* in a squash of spleen showing active schizont with multinucleation with thin capsule ×100; (**D**) *I. hoferi* schizont in spleen ×40; (**E**) The female nematode *Capillaria* with eggs ×40. (**F**) *Capillaria* egg with the plug at both poles ×100.

Microscopic examination of the internal parasites reveals different stages of the Mesomycetozoea *Ichthyophonus hoferi* (Figure 2). The resting spore/schizont in squash of kidney tissue with fibrotic capsule was observed (Figure 2A,B). Also, in a squash of spleen tissue, active schizont of *I. hoferi* with multinucleation with thin capsule was detected (Figure 2C,D). Intestinal examination shows the female nematode *Capillaria* with eggs internally (Figure 2E) with the characteristic double-operculated eggs with a plug at both poles (Figure 2F).

The overall parasite prevalence is 27.02% in all examined fish as shown in Table 2. Males show a higher rate of infection than females with a rate of 90%. In addition, the highest incidence of infection (19 Parasites) which represented 63.33% is found in fish with the weight class category II (110–200 g). Furthermore, fish with the weight categories I and IV recorded no parasitic infection. According to the fish length, the fish length categories I and III showed no infection, in contrary to the length class category II (18–28 cm) is the only range of fish length with parasitic infestation.

Table 3 shows the overall incidence and prevalence of parasites among fish assemblage. The overall prevalence of ectoparasites is 22.52% (25 out of 111) while endoparasites prevalence is 4.5% (5 out of 111). The prevalence of ectoparasitic infestation is 83.3% among infected fish and the parasitic infection included three protozoans infesting gills and skin; *T. truttae* with 10% infection rate, *T. heterodentata* and *I. multifiliis* with 3.3% infection rate per each. While the monogenean *Dactylogyrus* records the highest prevalence among infected fish assemblage with 60% infection rate of fish gills and skin. The monogenean (*Cichlidogyrus tilapiae*) infects the fish gills with 6.7% infection rate as shown in Table 3.

Internal infection in tilapias records Mesomycetozoea (*I. hoferi*) with 3.3% infection rate in the kidney and spleen. In addition, one fish sample with the coccidian infestation (mostly eimeriid) from the intestine is recorded. The intestine of the inspected fish shows 10% infection rate with the nematode (*Capillaria* spp.). Co-infection is commonly recorded between the protozoans *Trichodina,* the nematode (*Capillaria* spp.), the monogeneans, and the Mesomycetozoea (*I. hoferi*) during inspection (Table 3).

Tilapia males tend to attract parasitic infestation than females and eventually have more coinfection (Table 4 and Table 5). Tilapia at weight category II (110–200 g) is found to be the most appropriate body weight with high infestation with monogeneans followed by class III (210–300 g). The body length (class II) is the only length with infestation in all examined fish.

### 3.3. Haematological and Biochemical Analysis

#### 3.3.1. Haematological Parameters

Comparing infected and non-infected tilapias, the infected tilapias show a highly significant increase in white blood cells count compared to non-infected ones (*p* < 0.0001). Differential leukocyte count of neutrophils, lymphocytes, and basophil exhibits a non-significant increase (*p* = 0.2768, *p* = 0.2250, and *p* = 0.9547, respectively) between the two inspected tilapias groups. While monocytes, and eosinophil are significantly increased in the infected tilapias as compared to the non-infected one (*p* < 0.0001) (Table 6).

There is significant decrease in infected tilapias in RBCs, HGB, HCT, MCV, MCH, and MCHC (*p* < 0.0001, *p* = 0.0048, *p* = 0.0074, *p* < 0.0001, *p* = 0.0005, *p* = 0.0001, respectively). Platelet count shows a highly significant increase in infected fish compared to the non-infected fish (*p* < 0.0001) (Table 6).

#### 3.3.2. Biochemical Parameters

**Figure 3:**
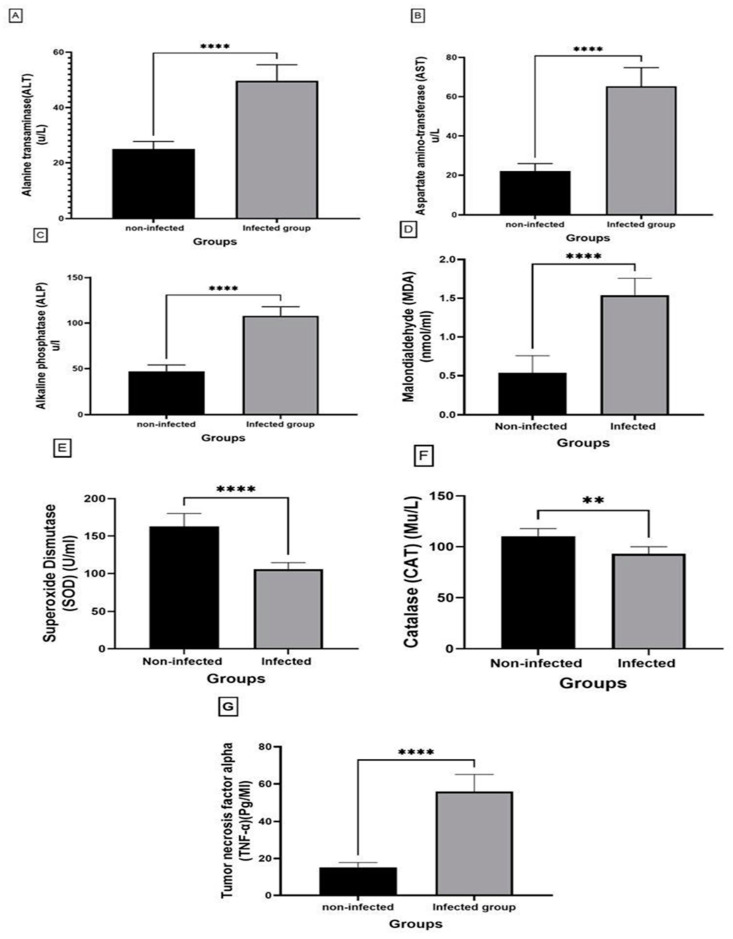
Biochemical analyses of non-infected and infected Nile Tilapia fish. (**A**) ALT, (**B**), AST, (**C**), ALP, (**D**), MDA, (**E**), SOD, (**F**), CAT, and (**G**), TNF-α. Data expressed as mean ± SD, N = 6 for each group. Significant difference between values of the two groups: ** (*p* < 0.01), **** (*p* < 0.0001).

Infected tilapias show a significant increase (*p* < 0.0001) in liver enzymes ALT, AST, and ALP as compared to the non-infected fish (Figure 3). There is a remarkably significant lowering (*p* < 0.0001, *p* < 0.05, respectively) in the antioxidants SOD and CAT activities with a highly significant increase in the oxidative stress marker MDA and the inflammatory cytokine TNFα in the infected fish relative to non-infected fish (*p* < 0.0001).

### 3.4. Histological Studies

#### 3.4.1. Liver and Hepatopancreas

**Figure 4:**
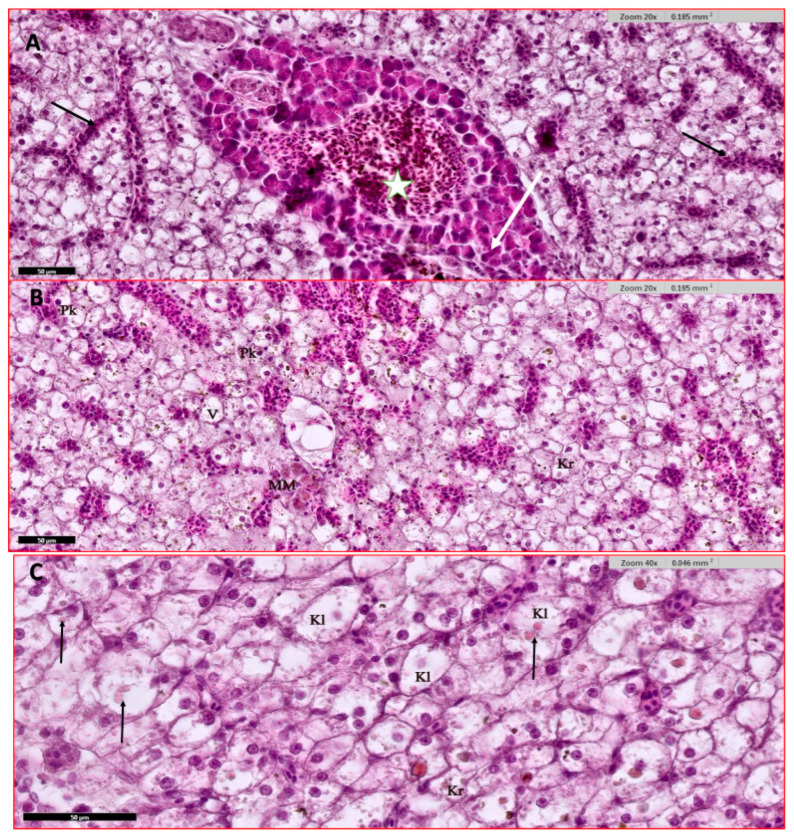
Histological section of infected Nile tilapia (*O. niloticus*) liver and hepatopancreas. (**A**) Liver diffusely dispersed with islets of exocrine pancreatic tissue containing secreting granules (white arrow) and showing severe hydropic degeneration of liver cells with signet-ring-like nucleus, ballooning of liver cells, severe liver cells congestion at sinusoidal spaces (black arrows) and blood vessels (star); (**B**) hepatopancreas showing severe sinusoidal spaces congestion, hydropic degeneration, vacuolation (V), melanomacrophages centers (MM), high hemosiderin granules deposition and variable cell injury at nucleus as pyknosis (Pk), kayrorrhexis (Kr); (**C**) hepatopancreas showing variable cell injury; hydropic degeneration, nucleus kayrorrhexis (Kr), nucleus karyolysis (Kl), intracytoplasmic eosinophilic inclusion and ballooning of cells containing these inclusions (arrows). H and E stain.

The liver of infected fish shows diffusely dispersed islets of exocrine pancreatic tissue scattered in the livers. The liver cells show severe hydropic degeneration and severe congestion in sinusoidal spaces and at blood vessels. Also, vacuolation near congested sinusoids is noted (Figure 4A). Ballooning and vacuolation of most liver cells are also seen near congested zones with signet-ring-like nucleus (Figure 4A,B). Melanomacrophages center (MM), high hemosiderin granules deposition (Figure 4B). Variable cell injury at the nucleus level is noted as pyknosis, karyorrhexis, and karyolysis with common intracytoplasmic eosinophilic inclusion and ballooning of these cells (Figure 4B,C).

#### 3.4.2. Gills

**Figure 5:**
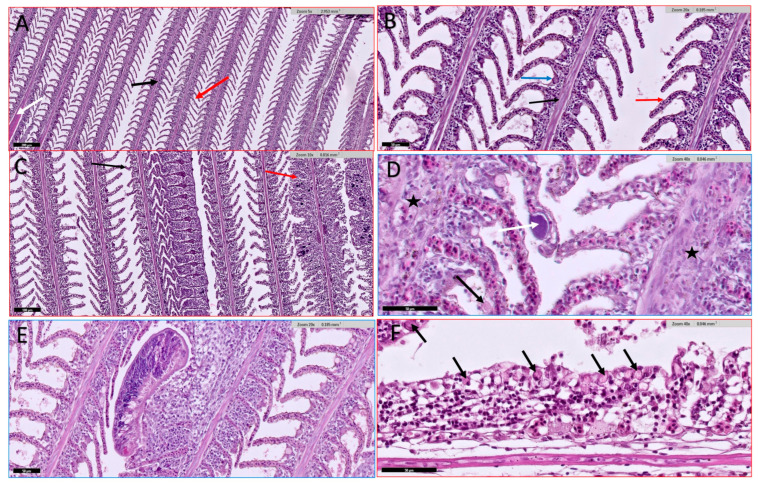
Histological section of Nile tilapia (*O. niloticus*) gills. (**A**) normal fish gills with normal primary lamella (black arrow), supported by filament cartilage (white arrow) and paired secondary lamella on each side of the primary lamellae (red arrow); (**B**) normal fish gills showing primary lamella (black arrow), secondary lamella (red arrow), and mucous cell (blue arrow); (**C**) infected fish gills showing fusion of secondary lamellae at their distal part (black arrow), swollen tip (red arrow) with necrotic cells replacing normal gill cells due to monogenean infection; (**D**) infected fish gills showing swollen primary lamellae (star) with epitheliocyst at the distal secondary lamella (white arrow), *Trichodina* infection (black arrow); (**E**) infected gills showing monogenean trematode attached to the gills causing secondary lamellae inflammation, swollen and fusion; (**F**) infected fish gills showing secondary lamellar erosion due to monogenean infection and rodlet cells (black arrows) activation in response to infection. H and E stain.

Examined non-infected fish have eight-gill arches with primary lamellae with many thin respiratory secondary lamellae (Figure 5A,B). Infected fish’s gills show inflammation and fusion of secondary lamellae, swollen tips (club-shaped), multifocal basophilic spots of necrotic cells replacing normal gill cells, blood vessel congestion, epithelia lifting, and even gill filaments necrosis (Figure 5C–F). Also, epitheliocyst is a bacterial infection that is commonly noted as a secondary consequence of stressed gills with parasitic infections such as *Trichodina* and monogenean trematodes (Figure 5D,E). The severity of the pathological features related to the intensity of the infection, as with severe infection, gills experienced erosion of gills and necrosis with obvious rodlet cells activation (Figure 5F).

#### 3.4.3. Intestine

**Figure 6:**
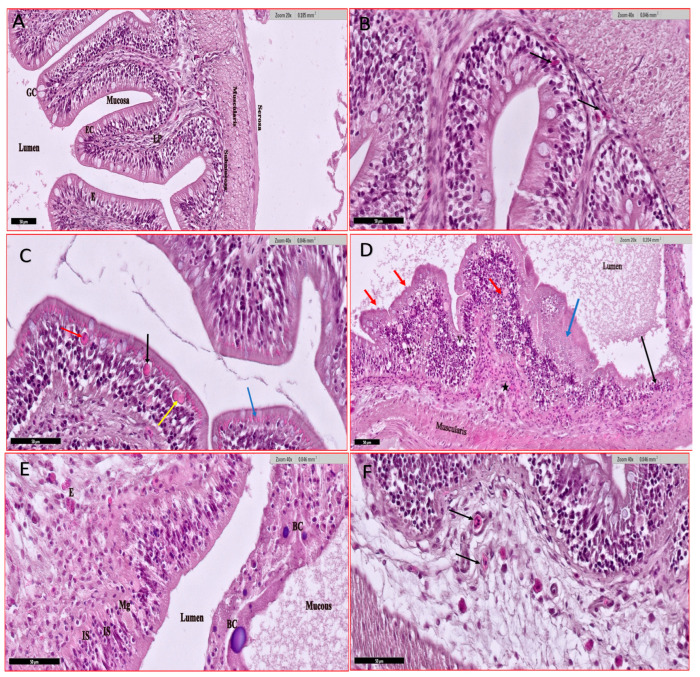
Histological section of Nile tilapia (*O. niloticus*) intestine H and E stain. (**A**) non-infected fish intestine showing normal architecture: the mucosa (the innermost layer), submucosa, and muscularis propria that are covered externally by serosa (the outermost layer). Layers of mucosa are the epithelium (E), and the lamina propria (LP). The epithelium is made up of enterocytes (EC), and goblet cells (GC); (**B**) non-infected fish intestine showing infiltration of mast cells/eosinophilic granular cells in the lamina propria (arrows); (**C**) infected small intestine with eimeriid coccidian parasites; immature gamont (Black Arrow), sporulated gamont (yellow arrow), macrogamont with central nucleus (Red Arrow) and secreting granules at the apical enterocytes (blue arrow); (**D**) infected fish intestine showing different stages of heavy coccidian (Eimeriidae) infestation (red arrows) causing obvious distortion and focal erosion of villi (black arrow) releasing different coccidian stages in lumen, hyperplasia of enterocytes and goblet cells (blue arrow), increased eosinophilic granules at lamina propria (asterisk), vacuolization of epithelia (V), increased intraepithelial lymphocytes and increased mucous secretion at the lumen); (**E**) infected fish small intestine with eimeriid coccidian showing immature schizont in rows at enterocytes (IS), sporulated microgamont (Mg), eosinophilic granules at the lamina propria (E), and excessive mucous in the lumen with cellular shading and bacterial cyst (BC); (**F**) infected fish small intestine with eimeriid coccidian showing mature schizont intraintestinal at the lamina propria containing merozoites in the parasitophorous vacuole membrane (arrows).

Uninfected fish’s intestine shows the normal architecture with mast cells/eosinophilic granules in the lamina propria (Figure 6A,B). Infected fish reveal variable pathological findings due to infection by severe coccidian (mostly eimeriid) infection (Figure 6C–F). The histological examination divulges activated exocrine pancreatic cells disperse at the outer periphery of the intestine at the haemolymphatic space between serosa and muscularis layers causing serosal and musculature hypertrophy. Abundant-secreting granules are found in the cytoplasm of infected enterocytes and are released to the gut lumen with intestinal debris (Figure 6C). Leucocyte infiltration at epithelia and granulomatous villi is observed (Figure 6D). The coccidian infection induced lesions and focal/complete erosion of villi releasing massive debris at the lumen (Figure 6D,E). Also, necrosis, vacuolation, and inflammatory infiltration are noted. The intestine shows different stages of infecting apical enterocytes and lamina propria (Figure 6F).

#### 3.4.4. Spleen

**Figure 7:**
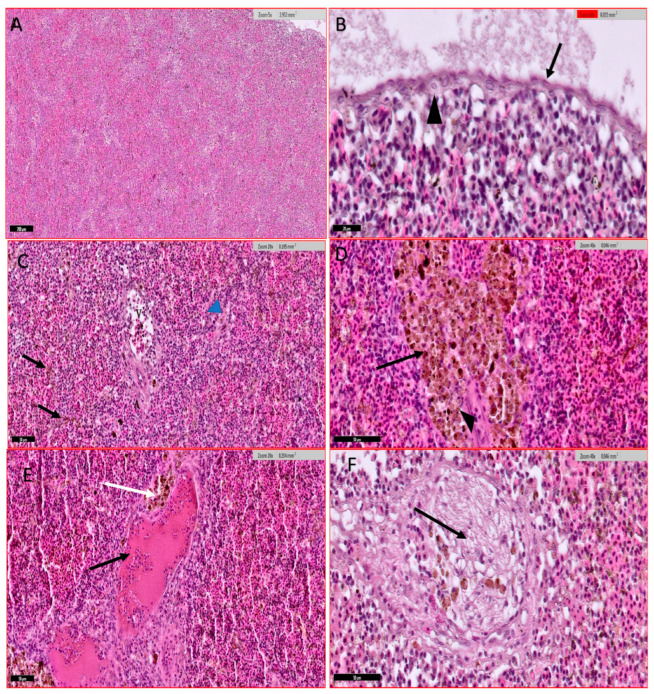
Histological section of Nile tilapia (*O. niloticus*) spleen H and E stain. (**A**) normal fish spleen architecture with the intermixed hematogenous red pulp (light pink color) including generally erythrocytes and lymphoid white pulp (light purple colour); (**B**) non-infected fish spleen covered externally with a thin capsule of single-layered squamous epithelium (arrow) and internally attached reticulocytes (arrowhead); (**C**) Infected fish spleen showing disseminated dark brown hemosiderin deposits due to the destruction of blood cells (arrow), supporting reticular fibres (arrowhead), arteriole (A), and venules (V); (**D**) Infected fish spleen showing brown melano-macrophage clusters in the spleen (arrow), mainly around splenic blood vessels (arrowhead); (**E**) Infected fish spleen showing vascular dilatation and congestion (black arrow) with melano-macrophage deposition (white arrow) around blood vessels and tissue necrosis; (**F**) Infected fish spleen showing blood vessel hyperplasia with subendothelial infiltration, luminal obliteration, and concentric accumulation of fibre bundles (arrow).

Histological examination of the spleen showed the spleen architecture where the white pulp and red pulp are intermixed (Figure 7A,B). The spleen showed disseminated dark brown hemosiderin deposits due to the destruction of blood cells and many brown melano-macrophage clusters mainly around splenic blood vessels (Figure 7C,D). The blood vessel encountered vascular dilatation, congestion, subendothelial infiltration, luminal obliteration, and concentric accumulation of fibre bundles (Figure 7E,F).

#### 3.4.5. Skin

**Figure 8:**
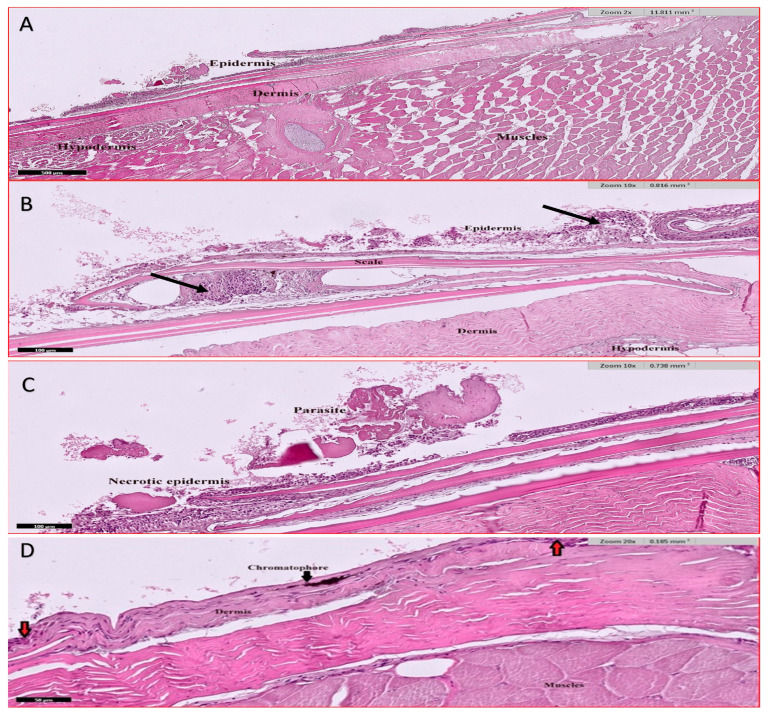
Histological section of Nile tilapia (*O. niloticus*) skin H and E stain. (**A**) fish skin showing basic 4 layers; epidermis, dermis, hypodermis, and muscles. The skin shows disorganized epithelium; (**B**) infected fish skin showing erosion of epidermis layer with ulcer and edema (arrows) due to parasitic infection; (**C**) infected fish skin showing ectoparasite with epidermis layer ulcer and necrosis; (**D**) infected fish skin showing complete erosion of both epidermis layer and scales. The dermis layer shows necrotic muscles (red arrow) at the top and inflamed and unorganized muscles at the bottom.

External environmental factors including parasitic infection result in variable pathological features. Low infection rates lead to slight erosion of the outer layer of the epidermis, leading to inflammation of the skin and edema (Figure 8A–C), while with intense infection, a complete erosion of the epidermis layer with its scales is noted exposing dermis to the external environment causing swelling and inflammation of the skin (Figure 8D).

### 3.5. Analysis of Heavy Metal in Fish Tissue

**Figure 9:**
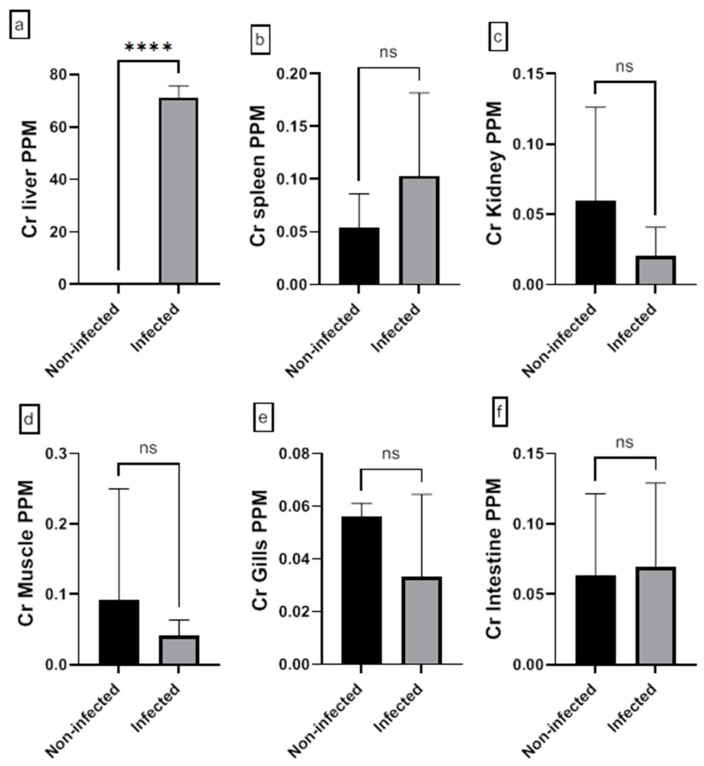
(**a**–**f**) Heavy metals (Cr) residues in different tissues (muscles, gill, liver, kidney, intestine, and spleen) from non-infected and infected Nile Tilapia fish. PPM: part per million wet weights. ns: non-significant, **** (*p* < 0.0001).

**Figure 10:**
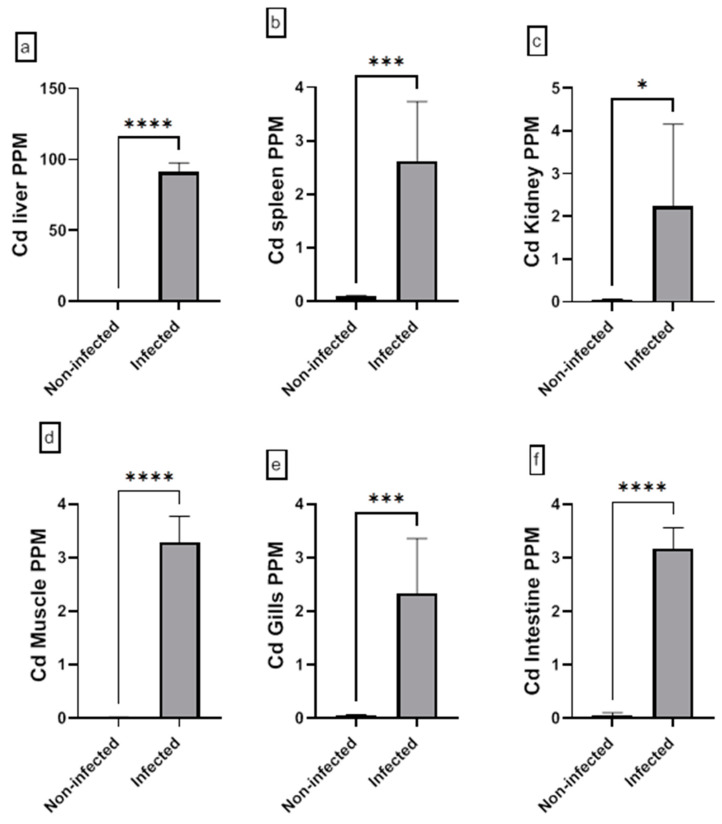
(**a**–**f**) Heavy metal (Cd) residues in different tissues (muscles, gill, liver, kidney, intestine, and spleen) from non-infected and infected Nile Tilapia fish. PPM: Part per million wet weights. * *p* < 0.05, *** *p* < 0.001, **** *p* < 0.0001.

**Figure 11:**
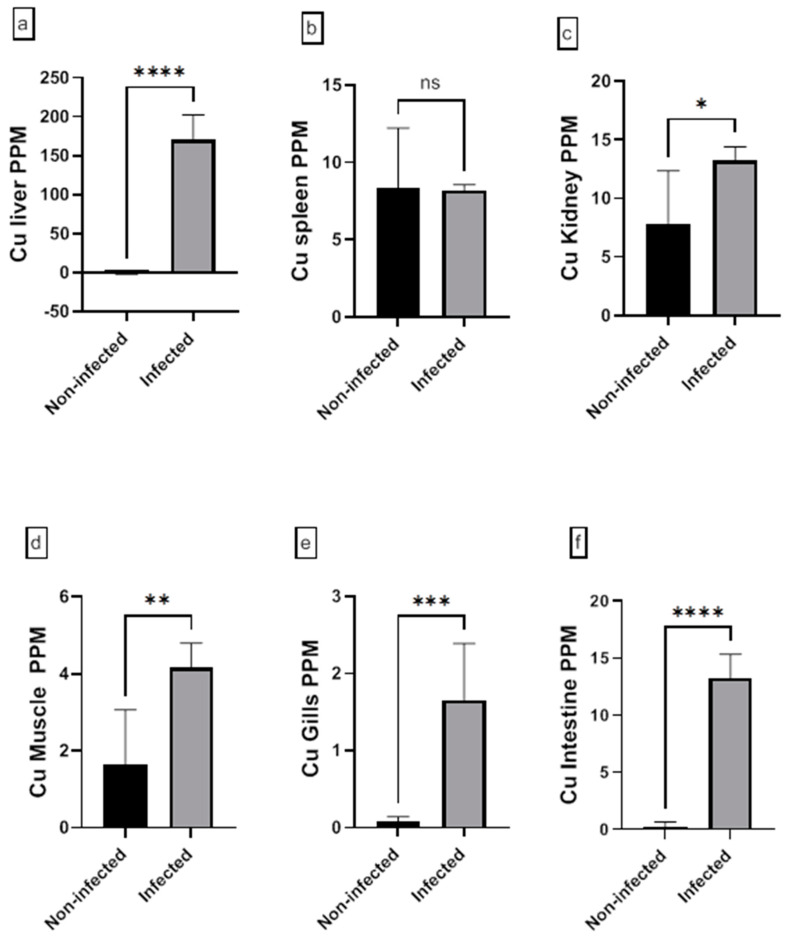
(**a**–**f**) Heavy metal (Cu) residues in different tissues (muscles, gill, liver, kidney, intestine, and spleen) from non-infected and infected Nile Tilapia fish. PPM: part per million wet weights. ns: non-significant, * *p* < 0.05, ** *p* < 0.01, *** *p* < 0.001, **** *p* < 0.0001.

**Figure 12:**
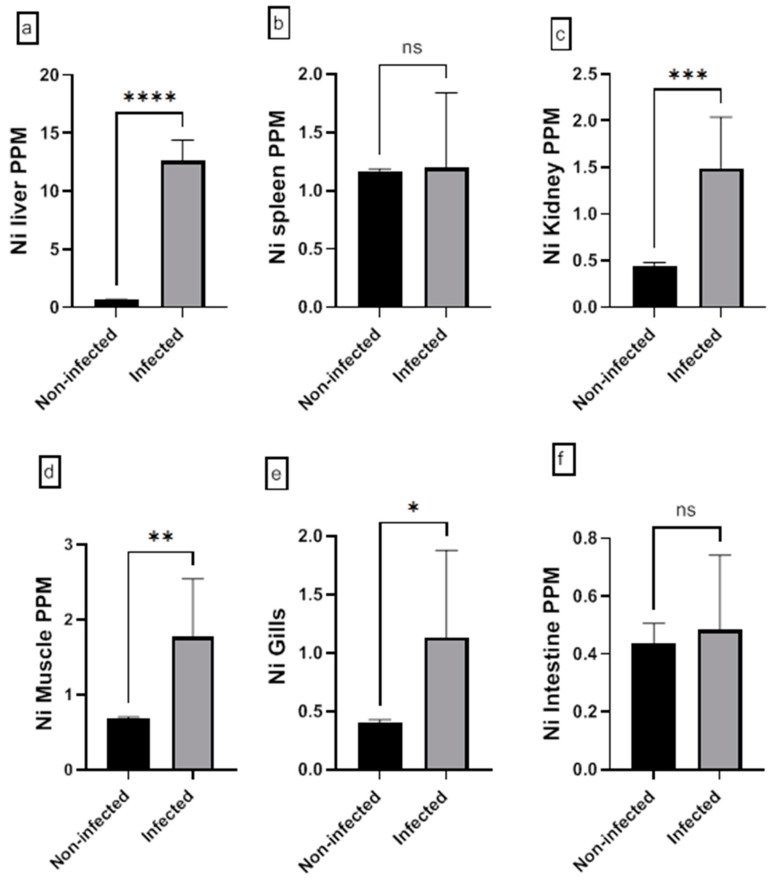
(**a**–**f**) Heavy metal (Ni) residues in different tissues (muscles, gill, liver, kidney, intestine, and spleen) of non-infected and infected Nile Tilapia fish. PPM: part per million wet weights. ns: non-significant, * *p* < 0.05, ** *p* < 0.01, *** *p* < 0.001, **** *p* < 0.0001.

**Figure 13:**
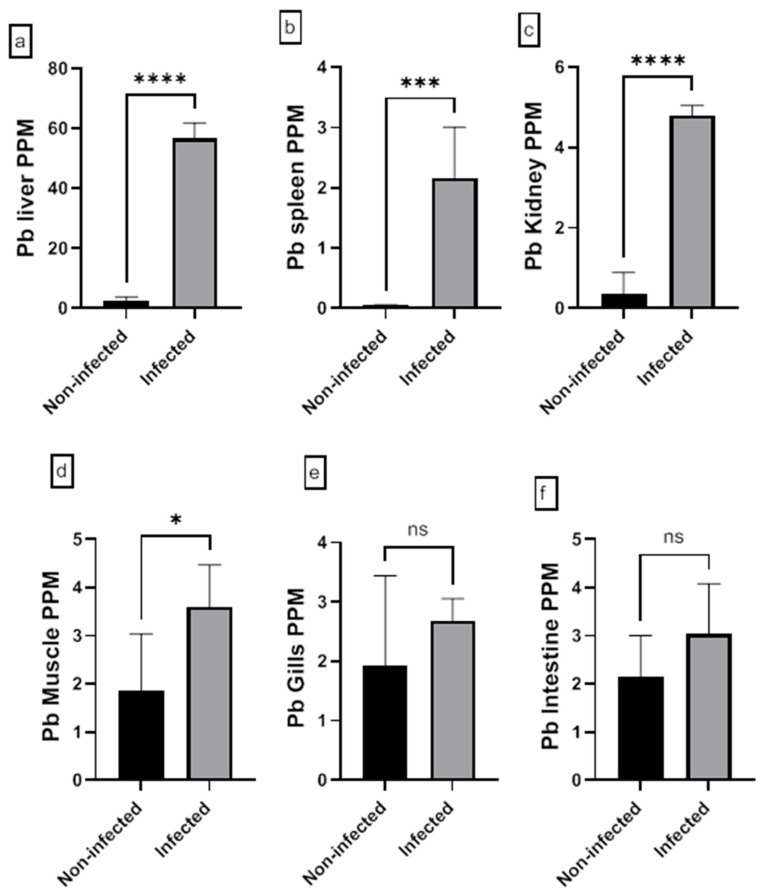
(**a**–**f**) Heavy metal (Pb) residues in different tissues (muscles, gill, liver, kidney, intestine, and spleen) of non-infected and infected Nile Tilapia fish. PPM: part per million wet weights. ns: non-significant, * *p* < 0.05, *** *p* < 0.001, **** *p* < 0.0001.

**Figure 14:**
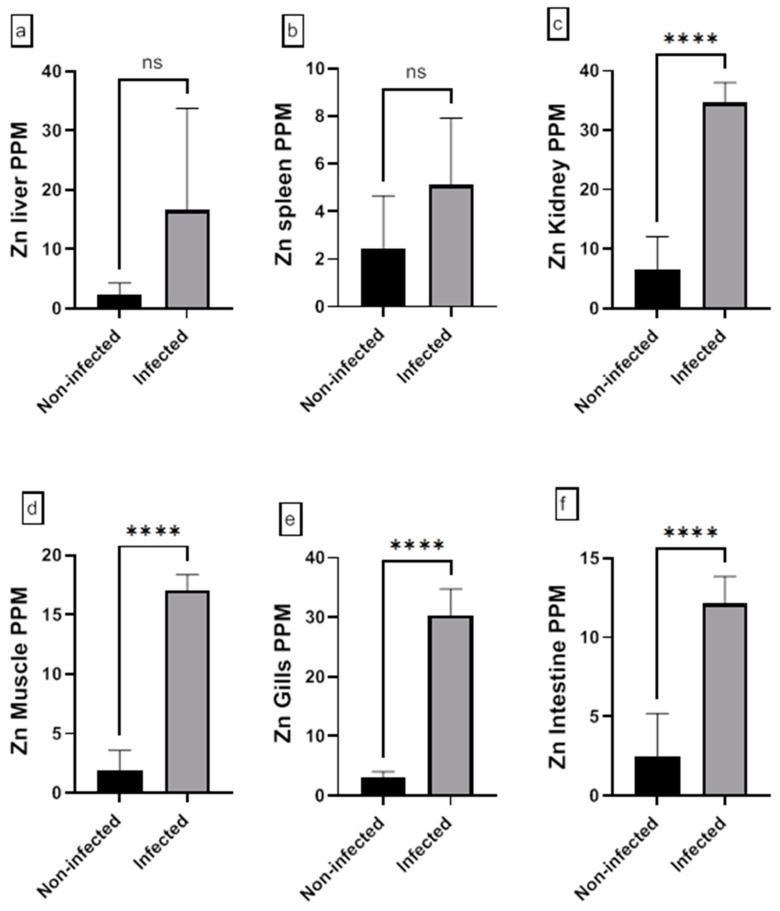
(**a**–**f**) Heavy metal (Zn) residues in different tissues (muscles, gill, liver, kidney, intestine, and spleen) of non-infected and infected Nile Tilapia fish. PPM: part per million wet weights. ns: non-significant, **** *p* < 0.0001.

The assessment of the heavy metal (Cr, Cd, Cu, Ni, Pb, and Zn) residues in fish musculature and non-edible tissues (gill, liver, kidney, intestine, and spleen) of non-infected and infected Nile tilapias is shown in Figure 9, Figure 10, Figure 11, Figure 12, Figure 13 and Figure 14 (see Supplementary S2 for numerical details). The Cr level shows a significant increase in the infected fish liver tissue compared to the non-infected ones. However, non-significant differences in Cr levels are reported in other tissues comparing infected fish to non-infected ones (Figure 9). There is a significant increase in Cd level at all tissues of infected fish compared to non-infected fish (Figure 10). In addition, Cu accumulation is significantly increased in all tissues of infected fish except in the spleen (Figure 11). Furthermore, there is a significant increase in Ni level in all infected fish tissue except in spleen and intestine tissues (Figure 12). The metal Pb is significantly highly accumulated in all infected fish tissues except in the gills and intestine compared to the non-infected fish (Figure 13). The Zn is significantly increased in all infected tilapias tissues except in the spleen as compared to the non-infected fish (Figure 14). More detailed multiple statistical comparisons between different tilapias tissues are shown in Supplementary S3.

Generally, heavy metal residues in infected Nile Tilapia fish tissues accumulate at a higher rate in the liver followed by the kidney then the intestine, gills, and finally muscles. The gills are contaminated with traces of heavy metals in the following order: Zn > Pb > Mn > Cr> Cu > Ni > Cd, whereas kidneys Zn > Cu > Pb > Cr> Ni > Cd. Splenic tissue of fishes showed marked higher concentrations of Cu and Zn. The levels of heavy metal residues in muscle tissues are significantly higher in accumulation of Zn, Cu, Pb, Cr, Ni, and Cd.

**Figure 15:**
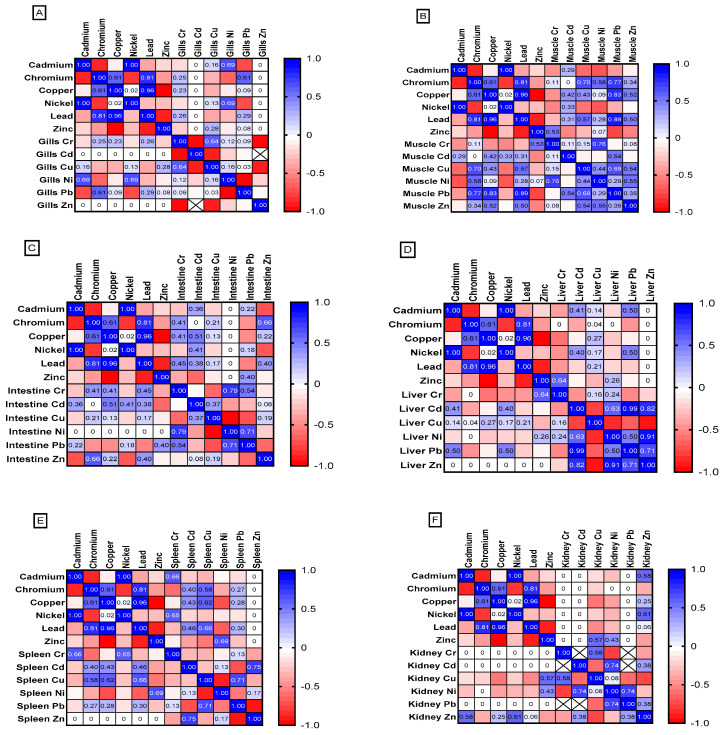
Pearson correlation coefficients between heavy metal levels in different fish tissue samples and heavy metals in aquaculture water. (**A**) gills, (**B**) muscle, (**C**) intestine, (**D**) liver, (**E**) spleen, and (**F**) kidney.

All studied fish tissues show higher rate of heavy metal accumulation than the surrounding water. Figure 15 shows the Pearson correlation between heavy metal levels in fish tissue samples and heavy metals in water. Gill tissue shows weak positive correlation in Cr, Cu and Pd bioaccumulation, while Ni show strong correlation (r = 0.69) (Figure 15A). Fish muscles show strong correlation for Cu accumulation (r = 0.43) and Pb accumulation (r = 0.89; *p* = 0.04) compared to the surrounding water and in contrary, weak positive correlation is detected for the heavy metals Cd and Cr (Figure 15B). While the intestine of tilapias shows moderate positive correlation for bioaccumulation of Ni, Cd, and Cr from water and weak positive for Cu (Figure 15C). The liver shows weak accumulation of Cd and Cu from surrounding waters (Figure 15D). The splenic tissues show moderate correlation in Pb accumulation (r = 0.3) and strong correlation for Cu accumulation (r = 0.62) from the surroundings (Figure 15E). However, Kidney tissue shows a non-significant correlation between the heavy metals’ concentrations within the kidney tissue and heavy metals concentration in water (Figure 15F).

## 4. Discussion

The current study is the first investigation to evaluate the food safety from cultured tilapias in Jeddah, Saudi Arabia. *Oreochromis niloticus*, the Nile tilapia, is a significant model for scientific research, economic development, food security, nutrition, and ecosystem management. Nile tilapia is one of the most extensively cultivated and eaten freshwater fish species globally [40]. The possible hazard-stressed conditions such as infection and anthropogenic pollution on farmed tilapias were assessed by investigation the physiological and histopathological alterations in relation to different factors such as fish sex, body weight, length, and heavy metals contamination.

Water quality plays a crucial role in fish farming, as it greatly impacts the health of the fish and influences the costs involved in bringing the product to market [41]. Because of tilapias broad pH tolerance range (6.2–8.3), and total ammonia tolerance (1.14 mg/L to 1.73 mg/L), Nile tilapia is now regarded as a commercially critical cultural species alongside other tilapia species [42,43]. The aquaculture water quality was investigated in the current culture system. The pH, salinity, and nitrates in were within the standard range of the universal recommended values [44,45,46]. Furthermore, the current estimated values of nitrates, nitrite, and chlorine were also determined to be within the typical accepted ranges as approved by other studies [29,45,47]. However, TDS, total hardness, total alkalinity, ammonia, and phosphate did not lie within WHO standard ranges [29,48] suggesting a lot of water-soluble salts and excessive water hardness. Thus, a suitable treatment is required to bring the water’s hardness down to an acceptable level. Additionally, in this study, high range of ammonia indicates stressed cultured fish and subsequent impaired immunity of these tilapias. Ammonia absorbed by fish enters their bloodstream, impacting blood properties and disrupts antioxidant balance, leading to oxidative damage [49].

Generally, parasite-induced diseases also pose a significant challenge for fish farmers due to their potential to reduce growth rates, increase mortality rates, and incur substantial financial losses [50]. Owing to the combined effects of heavy metal contamination, inadequate management practices, and bacterial diseases and parasites, fishery resources continue to experience a decline [51,52]. In the current investigation, the prevalence of ectoparasites and endoparasites infection was length and weight dependent as longer and heavier fish tended to be more susceptible to infection. The female category had the lowest rate of parasite infection, in contrary with males with 90% prevalence of infestation. This outcome agrees with [6,53,54,55] who reported that male fish tend to be more susceptible to infection due to hormonal factors. The length–weight relationship is significant in tilapias [56]. The present results revealed that a higher infection rate occurred at the weight class II (110–200 g) and the length class II (18–28 cm). The higher infection rate in adults is more likely related to the longer duration of time the older fish were exposed to the infectious agents in their environment [31,55,57].

In general, parasites can serve as indicators of environmental quality in fish farming systems [58]. Good pond water and proper management resulted in low rate of prevalence and intensity of parasites on *O. niloticus* from fishponds [59,60]. For instance, there is a strong relationship between ectoparasites and water quality and nutritional quality [61]. The ectoparasite infection prevalence in the present work was 83.3% from total infections which indicated high stock density [62] in addition to impaired water quality as high ammonia levels. Additionally, the present study indicated stressed cultured tilapias due to high monogenean infection rate as compared to previous studies in Indonesia and Ethiopia, which detected monogeneans by 15% and 4% of the examined fish, respectively [63,64].

The high monogeneans infestation reported in the present study was a crucial stress on the fish external. The fish skin and gills are the first line of defense against hazards in the surroundings. The architecture of fish skin is shaped to have multiple functions and one of these functions is to protect the fish from external hazards [65]. In the present study, the observed erosion and lesion of the fish skin implies also elevated rates of heavy metals exposure in addition to ectoparasitic infection. As, fish skin ulcers are one of the most well-recognized indicators of polluted or stressed aquatic environments [66]. Furthermore, in this investigation, the infected gills showed variable pathological features, ranging from mild inflammation to severe erosion of gill lamellae, that are mainly caused by high rate of monogeneans infections and high ammonia levels in the water. Oxidative damage from ammonia exposure is strongly linked to physiological toxicity in fish and gill tissue damage and swollen [49]. In the present work, such scenario is likely to cause impaired respiration in tilapias particularly infected ones that were noted to have considerably lower RBC and Hb counts than non-infected ones. As haematocrit percentage, haemoglobin rate, and erythrocyte count are indicators for the oxygen transportation capability of fish [67].

In the current investigation, ecto- and endoparasitic coinfection was commonly noted, and the common well-being of the fish was controlled by the severity and intensity of the infection. As multiple parasitic infection has additive, antagonistic, and synergistic effects between them [68,69], these interactions impact the host’s immune capability to overcome other infections and the rate of host survival [70,71]. In the present study, epitheliolytic of infected gill triggered by pathogenic intracellular bacteria was observed as a secondary consequence of heavy ectoparasitic infection. At aquaculture systems, bacterial fish infections are considered one of the highly significant problems facing these systems [72] that may lead to respiratory agony and death, particularly in cultured and juvenile fish [73,74]. The inspected infected fish gills in the present study showed also activation of rodlet cells that play a vital role in fighting bacterial infection. As rodlet cells are associated with the secretion of the peptide piscidin which has a strong antimicrobial activity [75,76].

Contamination by eimeriid coccidia is common in cichlid fish and it is a main cause of intestinal damage due to the rupture of the epithelium by the escaping merozoites and oocysts [6]. The current examination of the fish’s intestines divulged severe coccidian and nematode infections. Such infections resulted in broad inflammatory diseases as activated lymphocyte production, severe erosion, and necrosis of intestinal epithelium that played a main role in increasing intestinal bacterial contagion. As fish intestinal parasites associated with inflammatory diseases diminish fish immunity and expose fish to further bacterial infection that may result in inadequate growth, higher vulnerability to pathogens, less fitness and even direct mortalities [77,78]. Generally, parasitic infection with bacterial contamination pose a possible hazard for consumers and possible infection transmission neighborhood and over local market [79].

In the present study, all examined fish tissues showed residues of heavy metals with higher bioconcentrations in infected fish. Heavy metals are crucial markers of fish health [80]. For example, in the present investigation, the high Pb and Cu accumulations in fish muscle pose a health risk for the fish population and an ultimate threat to humans. Such high metal bioaccumulation in infected fish is mainly due to parasitic infections that serve as metal sinks for their fish host [81,82]. In the present study, high accumulation of heavy metals in the liver caused sever tissue damage that resulted in eosinophilic inclusions. The eosinophilic inclusions in the liver of the infected fish suggest either high internal pressure in the sinusoids or increased permeability of the hepatic cell membrane [83]. Both high level of heavy metals pollution, water ammonia and parasitic infections are effective stimulants to the production of ALT, AST and ALP values due to hepatic cells damage [84,85]. The current investigation revealed that infected *O. niloticus* had higher AST, ALT, ALP and MDA values and had lower SOD and CAT activities compared to non-infected ones.

Melanomacrophages (MM) are macrophage-like cells in hemo-lymphopoietic organs as liver and spleen that digest endogenous and exogenous material [86,87]. Unlike other vertebrates, fish have humoral immunity with MM instead of germinal centers [88]. In this study, histological examination showed activation of MM in the liver and an evident increase of MM clusters in spleen. Also, in the current investigation, a substantial difference in WBCs values was noted between infected and non-infected fish indicating active fish defense against different stressors in the present study. Also, infected fish had a significantly higher platelet count than non-infected fish. Such high platelet count indicates the effectiveness of the cellular immune system as a defense mechanism against parasites [85,89]. In the present investigation, a noteworthy elevation in TNF-α was observed in infected fish. TNF-α is an informative biomarker of oxidative cell damage [90,91]. The innate and acquired immunity are regulated by TNF-α, an essential pro-inflammatory cytokine that controls the inflammatory response at the early stages of infection and activate phagocytes and macrophages, which in turn enhanced the activity of destroying microorganisms [92,93].

Generally, the study indicates a direct correlation between heavy metal pollution and parasitic infestation that eventually impacted fish health and overall fish yield and pose a significant concern about adequate food safety in Jeddah city.

## 5. Conclusions

The most significant aquatic species that can accumulate heavy metals in their organs is the fish. The parasite impedes fish defense mechanisms, intensifying the toxic effects of heavy metals that have a detrimental impact on fish’s physiological homeostasis and the tissues health. The present study revealed the significance of parasitic infections and heavy metal pollution in aquaculture via synergistic effects that severely impact fish health and overall productivity. The study showed that parasitic infections and heavy metal pollution can lead to increased mortality, and heightened susceptibility to other diseases and even death. The study suggested probable consequences of consumption of parasitized fish that is also contaminated with heavy metals that can lead to serious health issues in humans.

## Figures and Tables

**Table 1:** Water quality parameters and heavy metal concentrations of the water collected during the study period. Each reading represents (mean ± SD).

**Water Quality Parameters**		
**Water Parameters (*n* = 6)**	**Mean ± SD**	**Standard Limits by WHO Guidelines**
pH (unit)	6.57 ± 0.22	5.5 to 9.5
Salinity (g/L)	0.88 ± 0.55	0.5 to 2.5
Electrical Conductivity (µS/cm)	1160 ± 520.5	100 to 2000
Total Dissolve Solids (mg/L)	777.5 ± 347.1 *	<400
Ammonia (mg/L)	6.83 ± 1.51 *	1.5
Nitrate (mg/L)	1.93 ± 1.17	0.2 to 219
Nitrite (mg/L)	0.02 ± 0.01	3
Total Alkalinity (mg/L)	77.00 ± 20.63 *	20
Total Hardness (mg/L)	291.7 ± 256.0 *	50–100
Phosphate (mg/L)	0.68 ± 0.24 *	0.4–0.5
Sulfate (mg/L)	72.92 ± 71.31	No health-based guideline value
Chlorine (mg/L)	0.13 ± 0.12	5
**Water heavy metal concentrations**		
**Heavy metal (PPM)** **(*n* = 6)**	**Mean ±SD**	**Standard limits by WHO guidelines**
Cr	0.37 ± 0.04 *	0.05
Cd	0.08 ± 0.04 *	0.005
Cu	1.11 ± 0.08	1.5
Ni	0.35 ± 0.19 *	0.07
Pb	0.427 ± 0.29 *	0.05
Zn	4.18 ± 1.47	5.0

(*) An increase from the global standard; n: number of samples; PPM: part per million.

**Table 2:** Rate of infection in Nile tilapia with different variables (sex, length, and weight).

Variable	Non-Infected (*n* = 81)	Infected (*n* = 30)	*p*-Value
Gender			
No. Male	51 (62.96%)	27 (90%)	*p* = 0.0051
No. Female	30 (37.04%)	3 (10%)	
Weight (g)			
Total Average	216.9 ± 65.52	157.0 ± 62.71	*p* < 0.0001
Male	218.2 ± 66.56	161.9 ± 64.33 ^a^	*p* = 0.0004 ^a^
Female	214.8 ± 64.77	113.3 ± 5.77 ^b^	*p* = 0.0112 ^b^
Weight Classes (g)			
I (70–100 g)	3 (3.70%)	0	*p* = 0.67
II (110–200 g)	46 (56.79%)	21 (70%)	
III (210–300 g)	28 (34.57%)	9 (30%)	
IV (>310 g)	4 (4.94%)	0	
Length (cm)			
Total Average	23.65 ± 2.935	20.72 ± 1.893	*p* < 0.0001
Male	23.61 ± 2.505	20 ± 1 ^a^	*p* < 0.0001 ^a^
Female	23.73 ± 3.596	20.17 ± 1.893 ^b^	*p* = 0.0256 ^b^
Length classes (cm)			
I (<18 cm)	1 (1.23%)	0	*p* = 0.6858
II (18–28 cm)	79 (97.53%)	30 (100%)	
III (>28 cm)	1 (1.23%)	0	

^a^ infected versus non-infected males; ^b^ infected versus non-infected females.

**Table 3:** Overall incidence and prevalence of parasites in infected Nile tilapia.

Parasite	Site of Infection	Incidence (*n* = 30)	Prevalence (%)
Protozoan (*Trichodina truttae*)	Skin/Gills	3	10
Protozoan (*Trichodina heterodentata)*	Skin	1	3.3
Protozoan *(Ichthyophthirius multifiliis)*	Skin	1	3.3
Monogenean (*Dactylogyrus* spp.)	Skin/Gills	18	60
Monogenean *(Cichlidogyrus tilapiae*)	Gills	2	6.7
Mesomycetozoea *(Icthyophonus hoferi)*	Kidney/Spleen	1	3.3
Coccidian protozoa	Intestine	1	3.3
Nematode (*Capillaria* spp.)	Intestine	3	10

**Table 4:** Incidence of external parasites in Nile tilapia in relation to sex, weight, and length.

	Trematode (Monogeneans)		Protozoa	
	*Dactylogyrus* spp.	*Cichlidogyrus tilapiae*	*Trichodina* spp.	*Icthyophthirius multifiliis*
Gender				
Male	16	2	3	1
Female	2	0	1	0
Weight (g)				
I (70–100 g)	0	0	0	0
II (110–200 g)	10	2	2	1
III (210–300 g)	8	0	2	0
Length (cm)				
I (<18 cm)	0	0	0	0
II (18–28 cm)	18	2	4	1
III (>28 cm)	0	0	0	0

**Table 5:** Incidence of internal parasites in Nile tilapia in relation to sex, weight, and length.

	Mesomycetozoea	Protozoa	Nematodes
	*Ichthyophonus hoferi*	Coccidian	*Capillaria* spp.
Gender			
Male	1	1	3
Female	0	0	0
Weight (g)			
70–100 g	0	0	0
110–200 g	1	1	3
210–300 g	0	0	0
Length (cm)			
<18 cm	0	0	0
18–28 cm	1	1	3
>28 cm	0	0	0

**Table 6:** Haematological analyses of infected and non-infected tilapias.

Parameter	Non-Infected Fish	Infected Fish	*p*-Value
WBC (10^3^/uL)	12.96± 2.675	33.17 ± 1.260	****
Neutrophils (10^3^/uL)	1.66 ± 0.332	1.85 ± 0.249	ns
Lymphocytes (10^3^/uL)	6.80 ± 0.194	7.23 ± 0.787	ns
Monocytes 10^3^/uL	0.566 ± 0.533	2.42 ± 0.388	****
Eosinophils (10^3^/uL)	0.008 ± 0.011	0.48 ± 0.1909	***
Basophil (10^3^/uL)	0.821 ± 0.575	0.84 ± 0.724	ns
RBC (10^6^/uL)	5.23 ± 0.430	1.470 ± 0.287	****
HGB (g/dL)	8.367 ± 1.527	5.700 ± 0.971	**
HCT (%)	39.80 ± 1.230	32.05 ± 5.542	**
MCV (fL)	185.8 ± 2.872	166.8 ± 5.095	****
MCH (pg)	36.87 ± 2.333	30.75 ± 1.861	***
MCHC (g/dL)	24.37 ± 2.002	18.58 ± 1.132	****
PLT count (10^3^/uL)	16.00 ± 2.966	27.00 ± 1.789	****

(WBC) white blood cells, (RBCs), haemoglobin (HGB), haematocrit (HCT), mean corpuscular volume (MCV), mean corpuscular haemoglobin (MCH), mean corpuscular haemoglobin concentration (MCHC), Platelet (PLT). Significant difference between values of the two groups: ** (*p* < 0.01), *** (*p* < 0.001), **** (*p* < 0.0001), ns (non-significant).

## Data Availability

All data generated or analysed during this study are included in this published article and any further requirements for raw data are welcomed.

## Supplementary Materials

The following supporting information can be downloaded at: https://www.mdpi.com/article/10.3390/ani15010039/s1. S1: Pearson Correlation Coefficients between physio-chemical parameters, water heavy metals and fish tissue heavy metals. S2: Heavy metal tissue concentrations in non-infected and infected Nile Tilapia fish samples. S3: The Two-way ANOVA followed by Tukey’s multiple comparisons test to quantify the statistical difference between different organs in infected Nile Tilapia fish for each heavy metal level.
