# Supplementary material for: Food Safety: Pathological and Biochemical Responses of Nile Tilapia (Oreochromis niloticus) to Parasitological Infestation and Heavy Metals Pollution in Aquaculture System, Jeddah, Saudi Arabia
Source: Animals (Basel). 2024 Dec 27;15(1):39. doi: 10.3390/ani15010039 (PMC11718979; doi:10.3390/ani15010039)
Supplement: Supplementary file 1 [file animals-15-00039-s001.zip › animals-3358615-supplementary.pdf]

Supplementary S1

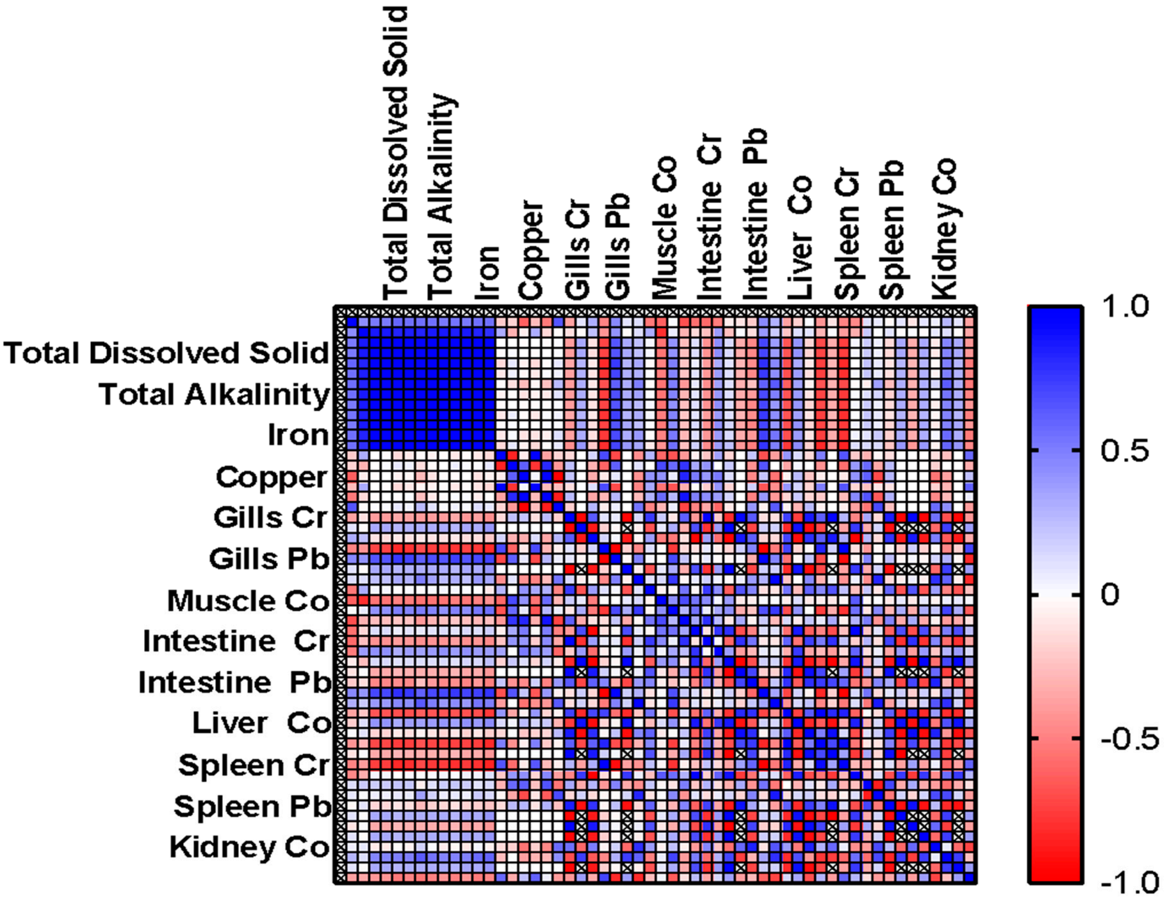

S1: Pearson Correlation Coefficients between physio-chemical parameters, water heavy metals and fish tissue heavy metals.

Supplementary S2

S2: Heavy metal tissue concentrations in non-infected and infected Nile Tilapia fish samples.

		Cr	Cd	Cu	Ni	Pb	Zn
**Gills**	**Non-infected**	0.05±0.004	0.04±0.024	0.07±0.064	0.39±0.027	1.92±1.51	2.94±1.09
	**infected**	0.03±0.03	2.34±1.01	1.65±0.73	1.13±0.74	2.68±0.36	30.33±4.42
**Muscle**	**Non-infected**	0.09±0.15	0.01±0.007	1.65±1.40	0.67±0.02	1.87±1.17	1.91±1.67
	**infected**	0.04±0.02	3.28±0.48	4.16±0.63	1.76±0.77	3.60±0.86	17.05±1.32
**Intestine**	**Non-infected**	0.06±0.05	0.065±0.03	0.24±0.41	0.43±0.07	2.14±0.85	2.48±2.70
	**infected**	0.07±0.06	3.16±0.39	13.25±2.09	0.48±0.25	3.04±1.04	12.14±1.70
**Liver**	**Non-infected**	0.64±0.02	0.06±0.04	-0.75 ±1.41	0.65±0.02	2.62±1.22	2.29±2.00
	**infected**	71.28±4.45	91.34±6.30	170.7±31.3	12.59±1.80	56.67±5.15	16.58±17.16
**Spleen**	**Non-infected**	0.05±0.03	0.10±0.005	8.36±3.88	1.17±0.01	0.04±0.01	2.45±2.18
	**infected**	2.62±1.11	0.10±0.07	8.18±0.38	1.20±0.64	2.16±0.84	5.10±2.80
**Kidney**	**Non-infected**	0.05±0.06	0.03±0.02	7.85±4.50	0.44±0.03	0.34±0.54	6.49±5.57
	**infected**	0.02±0.02	2.24±1.91	13.22±1.18	1.49±0.54	4.79±0.25	34.60±3.42
**FAO/WHO**		0.05	0.5	100	8.97	0.3	30

Data are represented as Mean ± SD. Key: Permissible maximum limit as cited by [31].

Supplementary S3

S3: The Two-way ANOVA followed by Tukey's multiple comparisons test to quantify the statistical difference between different organs in infected Nile Tilapia fish for each heavy metal level.

**Tukey's multiple comparisons test**	**Summary**	**Adjusted P Value**
**Cadmium**		
Gills vs. Muscle	ns	>0.9999
Gills vs. Intestine	ns	>0.9999
Gills vs. Liver	****	<0.0001
Gills vs. Spleen	ns	>0.9999
Gills vs. Kidney	ns	>0.9999
Muscle vs. Intestine	ns	>0.9999
Muscle vs. Liver	****	<0.0001
Muscle vs. Spleen	ns	>0.9999
Muscle vs. Kidney	ns	>0.9999
Intestine vs. Liver	****	<0.0001
Intestine vs. Spleen	ns	>0.9999
Intestine vs. Kidney	ns	>0.9999
Liver vs. Spleen	****	<0.0001
Liver vs. Kidney	****	<0.0001
Spleen vs. Kidney	ns	>0.9999
**Chromium**		
Gills vs. Muscle	ns	0.9998
Gills vs. Intestine	ns	>0.9999
Gills vs. Liver	****	<0.0001
Gills vs. Spleen	ns	>0.9999
Gills vs. Kidney	ns	>0.9999
Muscle vs. Intestine	ns	>0.9999
Muscle vs. Liver	****	<0.0001

Muscle vs. Spleen	ns	>0.9999
Muscle vs. Kidney	ns	0.9997
Intestine vs. Liver	****	<0.0001
Intestine vs. Spleen	ns	>0.9999
Intestine vs. Kidney	ns	0.9999
Liver vs. Spleen	****	<0.0001
Liver vs. Kidney	****	<0.0001
Spleen vs. Kidney	ns	>0.9999
**Copper**		
Gills vs. Muscle	ns	0.9828
Gills vs. Intestine	*	0.0206
Gills vs. Liver	****	<0.0001
Gills vs. Spleen	ns	0.4715
Gills vs. Kidney	ns	>0.9999
Muscle vs. Intestine	ns	0.1301
Muscle vs. Liver	****	<0.0001
Muscle vs. Spleen	ns	0.879
Muscle vs. Kidney	ns	0.9773
Intestine vs. Liver	****	<0.0001
Intestine vs. Spleen	ns	0.7308
Intestine vs. Kidney	*	0.018
Liver vs. Spleen	****	<0.0001
Liver vs. Kidney	****	<0.0001
Spleen vs. Kidney	ns	0.4431
**Nickel**		
Gills vs. Muscle	ns	>0.9999
Gills vs. Intestine	ns	>0.9999
Gills vs. Liver	*	0.0231
Gills vs. Spleen	*	0.0135
Gills vs. Kidney	ns	0.9997
Muscle vs. Intestine	ns	0.9993

Muscle vs. Liver	*	0.0383
Muscle vs. Spleen	*	0.0231
Muscle vs. Kidney	ns	>0.9999
Intestine vs. Liver	*	0.0133
Intestine vs. Spleen	**	0.0075
Intestine vs. Kidney	ns	0.9973
Liver vs. Spleen	ns	>0.9999
Liver vs. Kidney	ns	0.0519
Spleen vs. Kidney	*	0.0319
**Lead**		
Gills vs. Muscle	ns	0.9999
Gills vs. Intestine	ns	>0.9999
Gills vs. Liver	****	<0.0001
Gills vs. Spleen	ns	0.9985
Gills vs. Kidney	ns	0.9924
Muscle vs. Intestine	ns	>0.9999
Muscle vs. Liver	****	<0.0001
Muscle vs. Spleen	ns	0.986
Muscle vs. Kidney	ns	0.9995
Intestine vs. Liver	****	<0.0001
Intestine vs. Spleen	ns	0.9959
Intestine vs. Kidney	ns	0.9968
Liver vs. Spleen	****	<0.0001
Liver vs. Kidney	****	<0.0001
Spleen vs. Kidney	ns	0.9216
**Zinc**		
Gills vs. Muscle	**	0.0045
Gills vs. Intestine	****	<0.0001
Gills vs. Liver	**	0.0029
Gills vs. Spleen	****	<0.0001
Gills vs. Kidney	ns	0.8486

Muscle vs. Intestine	ns	0.7562
Muscle vs. Liver	ns	>0.9999
Muscle vs. Spleen	*	0.0152
Muscle vs. Kidney	****	<0.0001
Intestine vs. Liver	ns	0.8269
Intestine vs. Spleen	ns	0.3839
Intestine vs. Kidney	****	<0.0001
Liver vs. Spleen	*	0.0228
Liver vs. Kidney	****	<0.0001
Spleen vs. Kidney	****	<0.0001

ns means non-significant. * Significant difference between values of the two groups (<0.05), ** Significant difference between values of the two groups (P<0.01), and **** Significant difference between values of the two groups (P<0.0001).

Table S1: The two-way ANOVA followed by Tukey's multiple comparisons test to quantify the statistical difference between the heavy metals' levels in various in infected Nile Tilapia fish organ samples for each metal.

**Tukey's multiple comparisons test**	**Summary**	**Adjusted P Value**
**Gills**		
Cadmium vs. Chromium	ns	0.9883
Cadmium vs. Copper	ns	0.9978
Cadmium vs. Nickel	ns	0.9997
Cadmium vs. Lead	ns	0.978
Cadmium vs. Zinc	****	<0.0001
Chromium vs. Copper	ns	>0.9999
Chromium vs. Nickel	ns	0.9995
Chromium vs. Lead	ns	>0.9999
Chromium vs. Zinc	****	<0.0001
Copper vs. Nickel	ns	>0.9999
Copper vs. Lead	ns	0.9997
Copper vs. Zinc	****	<0.0001
Nickel vs. Lead	ns	0.9982

Nickel vs. Zinc	****	<0.0001
Lead vs. Zinc	****	<0.0001
**Muscle**		
Cadmium vs. Chromium	ns	0.9482
Cadmium vs. Copper	ns	0.8666
Cadmium vs. Nickel	ns	0.997
Cadmium vs. Lead	ns	0.9242
Cadmium vs. Zinc	****	<0.0001
Chromium vs. Copper	ns	0.9999
Chromium vs. Nickel	ns	0.9984
Chromium vs. Lead	ns	>0.9999
Chromium vs. Zinc	**	0.0028
Copper vs. Nickel	ns	0.986
Copper vs. Lead	ns	>0.9999
Copper vs. Zinc	**	0.0066
Nickel vs. Lead	ns	0.996
Nickel vs. Zinc	***	0.0006
Lead vs. Zinc	**	0.0038
**Intestine**		
Cadmium vs. Chromium	ns	0.9571
Cadmium vs. Copper	**	0.005
Cadmium vs. Nickel	ns	>0.9999
Cadmium vs. Lead	ns	0.9641
Cadmium vs. Zinc	*	0.0137
Chromium vs. Copper	ns	0.0667
Chromium vs. Nickel	ns	0.9769
Chromium vs. Lead	ns	>0.9999
Chromium vs. Zinc	ns	0.1392
Copper vs. Nickel	**	0.0074
Copper vs. Lead	ns	0.0609
Copper vs. Zinc	ns	0.9996

Nickel vs. Lead	ns	0.9814
Nickel vs. Zinc	*	0.0195
Lead vs. Zinc	ns	0.1286
**Liver**		
Cadmium vs. Chromium	****	<0.0001
Cadmium vs. Copper	****	<0.0001
Cadmium vs. Nickel	****	<0.0001
Cadmium vs. Lead	**	0.0012
Cadmium vs. Zinc	****	<0.0001
Chromium vs. Copper	****	<0.0001
Chromium vs. Nickel	****	<0.0001
Chromium vs. Lead	****	<0.0001
Chromium vs. Zinc	****	<0.0001
Copper vs. Nickel	****	<0.0001
Copper vs. Lead	****	<0.0001
Copper vs. Zinc	****	<0.0001
Nickel vs. Lead	****	<0.0001
Nickel vs. Zinc	ns	0.8824
Lead vs. Zinc	****	<0.0001
**Spleen**		
Cadmium vs. Chromium	ns	0.9825
Cadmium vs. Copper	ns	0.2331
Cadmium vs. Nickel	**	0.0053
Cadmium vs. Lead	ns	0.9997
Cadmium vs. Zinc	ns	0.7417
Chromium vs. Copper	ns	0.6464
Chromium vs. Nickel	*	0.0454
Chromium vs. Lead	ns	0.9988
Chromium vs. Zinc	ns	0.9837
Copper vs. Nickel	ns	0.7347
Copper vs. Lead	ns	0.3935

Copper vs. Zinc	ns	0.9584
Nickel vs. Lead	*	0.0143
Nickel vs. Zinc	ns	0.2278
Lead vs. Zinc	ns	0.8912
**Kidney**		
Cadmium vs. Chromium	ns	0.9901
Cadmium vs. Copper	ns	0.9986
Cadmium vs. Nickel	ns	0.9916
Cadmium vs. Lead	ns	0.7784
Cadmium vs. Zinc	****	<0.0001
Chromium vs. Copper	ns	>0.9999
Chromium vs. Nickel	ns	>0.9999
Chromium vs. Lead	ns	0.9818
Chromium vs. Zinc	****	<0.0001
Copper vs. Nickel	ns	>0.9999
Copper vs. Lead	ns	0.9443
Copper vs. Zinc	****	<0.0001
Nickel vs. Lead	ns	0.9791
Nickel vs. Zinc	****	<0.0001
Lead vs. Zinc	****	<0.0001

ns means non-significant. * Significant difference between values of the two groups (<0.05), ** Significant difference between values of the two groups (P<0.01), *** Significant difference between values of the two groups (P<0.001), and **** Significant difference between values of the two groups (P<0.0001).
